# Apicotomy as Treatment for Failure of Orthodontic Traction

**DOI:** 10.1155/2013/168232

**Published:** 2013-12-04

**Authors:** Leandro Berni Osório, Vilmar Antonio Ferrazzo, Geraldo Serpa, Kívia Linhares Ferrazzo

**Affiliations:** ^1^Stomatology Department, Pediatric Dentistry, School of Dentistry, Federal University of Santa Maria, 97015-370 Santa Maria, RS, Brazil; ^2^Department of Orthodontics, Pontificia Universidade Católica do Rio Grande do Sul, 90619-900 Porto Alegre, RS, Brazil; ^3^Stomatology Department, Orthodontics, School of Dentistry, Federal University of Santa Maria, 97015-370 Santa Maria, RS, Brazil; ^4^Stomatology Department, Radiology, School of Dentistry, Federal University of Santa Maria, 97015-370 Santa Maria, RS, Brazil; ^5^Oral Medicine and Oral Pathology, School of Dentistry, Franciscan University Center, 97015-370 Santa Maria, RS, Brazil

## Abstract

*Objective.* The purpose of this study was to present a case report that demonstrated primary failure in a tooth traction that was subsequently treated with apicotomy technique. *Case Report.* A 10-year-old girl had an impacted upper right canine with increased pericoronal space, which was apparent on a radiographic image. The right maxillary sinus showed an opacity suggesting sinusitis. The presumptive diagnosis was dentigerous cyst associated with maxillary sinus infection. The plan for treatment included treatment of the sinus infection and cystic lesion and orthodontic traction of the canine after surgical exposure and bonding of an orthodontic appliance. The surgical procedure, canine position, root dilaceration, and probably apical ankylosis acted in the primary failure of the orthodontic traction. Surgical apical cut of the displaced teeth was performed, and tooth position in the dental arch was possible, with a positive response to the pulp vitality test. *Conclusion.* Apicotomy is an effective technique to treat severe canine displacement and primary orthodontic traction failure of palatally displaced canines.

## 1. Introduction

Impacted teeth are common abnormalities of development and the prevalence ranges from 5% to 18% [1–3]. The maxillary permanent canine is the second most prevalent impacted tooth in the human dentition (8%–10%), after third molars [4, 5]. Girls are more often affected than boys [6], and palatally positioned canines are more frequent than buccally positioned ones, at a ratio of 3 : 1, respectively [7].

The displacement of an upper canine is determined by distinct etiopathogenesis. Lack of space in the dental arch, trauma, and loss of primary teeth as well as a genetic component are pointed out as etiological factors [8, 9]. The facial impaction of a maxillary permanent canine is usually more frequent in crowded dental arches, while palatal impaction usually shows no association with any particular malocclusion [7]. This fact contributes to the late recognition of the wrong position of these teeth and can be a determining factor in the failure of orthodontic movement of palatal-impacted canines. Sometimes, bring these teeth into the correct position represents a challenge [10].

Surgical access to a displaced tooth is the key factor in treatment success, and the intervention should be carefully planned. There are three main types of surgery that are possible for managing impacted teeth: extraction, exposure for spontaneous eruption, and exposure for orthodontic traction with bonding devices [6, 11, 12]. For orthodontic traction, the surgical techniques can still be divided in apically positioned flap, for buccally positioned canines, closed eruption technique, for canines in the middle of the alveolar bone, and tunnelization for palatal-impacted teeth [5, 13].

Becker et al. [14] classified the reasons for the failure of orthodontic traction at dependent factors of the patient, of the orthodontist, and of the surgeon. Patient-dependent factors are age, anatomical local factors, such as root anomalies, angle of tooth eruption, and associated complications, such as lack of compliance. Orthodontist-dependent factors can be identified as misdiagnosis of tooth position, improper directional force, and the use of inefficient appliances. Surgeon-dependent factors include the misdiagnosis of tooth position, exposing the wrong side, damage on the impacted tooth, injury to an adjacent tooth, and surgery without orthodontic planning.

In those cases in which the tooth has local factors contributing to lack of orthodontic movement, a new surgical intervention can be necessary. Ankylosis is often the main local cause for failure of the movement of the impacted tooth [14]. Mobilization of the ankylosed tooth with forceps is a technique used to help orthodontic movement, but, in some cases, the root anatomy is unfavorable, avoiding the tooth movement.

The apicotomy is a technique described in 1987 that aims to liberate the part of the tooth with root dilacerations or ankylosis, thus allowing the traction and eruption. The technique consists of surgical fracture of the apical portion of the root and may be indicated after failure of conservative techniques to promote the correct position of the canine [15, 16].

The purpose of this study was to present a case report that showed primary failure in the tooth traction and was treated using the apicotomy technique.

## 2. Case Report

This case report was submitted and approved by the Research Ethical Committee of the Franciscan University Center, under number CEP/UNIFRA 346.2010.2. A 10-year-old girl was referred to the Integrated Clinic of the Franciscan University Center, Santa Maria, Brazil, with the complaint of recurrent rhinorrhea that has not responded after two months of antibiotic therapy. Extra oral physical examination revealed a slight facial asymmetry with swelling over the right maxillary sinus and nasal obstruction. The intraoral examination showed the presence of a deciduous canine and a maxillary right first molar.

**Figure 1:**
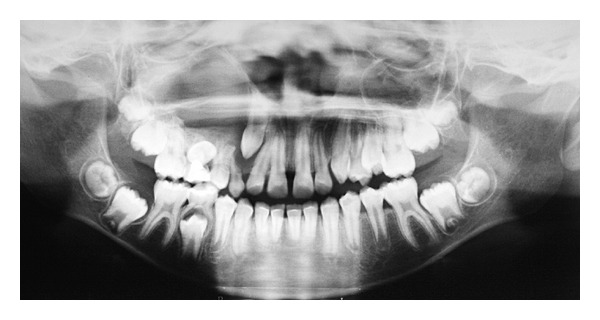
Initial panoramic radiography: retention of the upper right second premolar and upper right canine with increased pericoronal space, vertical impaction, and root dilaceration; root resorption of primary teeth; opacity of the right maxillary sinus.

**Figure 2:**
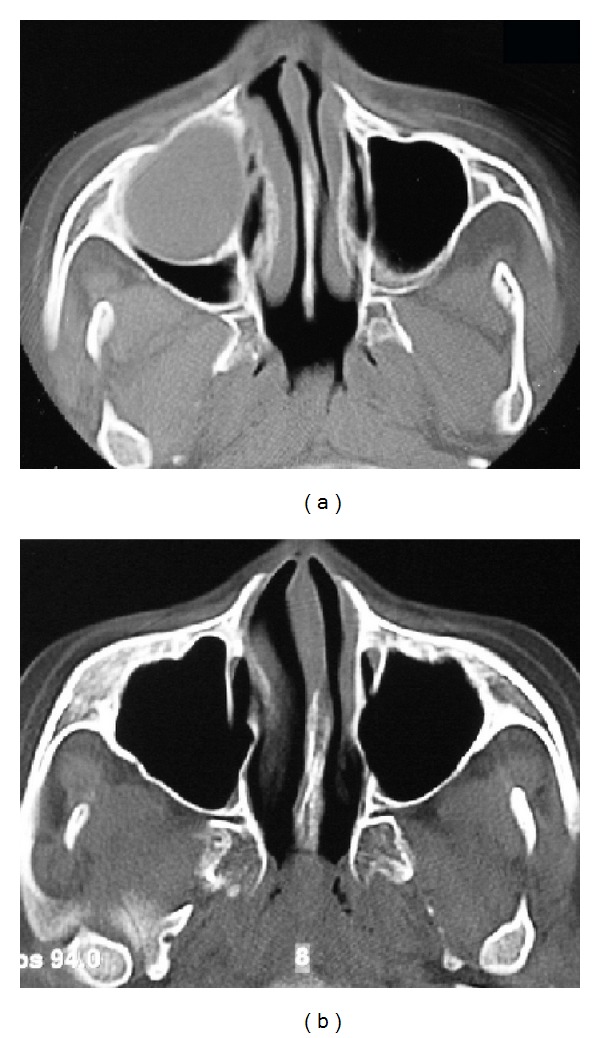
CT axial images showing opacity of the right maxillary sinus in the diagnostic phase (a); maxillary sinus cleared after 3 months of treatment (b).

The absence of primary teeth roots was identified in image exams (panoramic radiograph and computed tomography). Moreover, impactions of the upper right second premolar and of the upper right canine, associated with increased pericoronal space, were observed. The right maxillary sinus showed opacity (Figures 1 and 2(a)). The presumptive diagnosis was dentigerous cyst associated with maxillary sinus infection. Planning for this case has included the treatment of the sinus infection and of the cystic lesion, orthodontic traction of the canine after surgical exposure, and bonding of an orthodontic appliance to allow the traction of the canine.

The surgeon performed a Caldwell-Luc incision [17] under general anesthesia to access the right maxillary sinus. The cavity was washed with saline solution, and a fragment of the cystic capsule was obtained for histopathological examination. The cystic lesion caused a large amount of bone loss, and so, a conservative treatment was performed by surgical decompression, and a Penrose drain was inserted in the canine region. The primary teeth (canine and first molar) were extracted. The histopathological analysis revealed a stratified squamous epithelium of a few layers, confirming the dentigerous cyst hypothesis.

Amoxicillin with clavulanate potassium was used for 2 weeks. The drain was monitored for 3 months until there was no more secretion. At the time, no signs of sinus infection in imaging exams were observed (Figure 2(b)). After removing the drain, an attachment was bonded in the canine for orthodontic traction. Extractions of four first premolars were planned, but initially only the right maxillary premolar has been removed.

**Figure 3:**
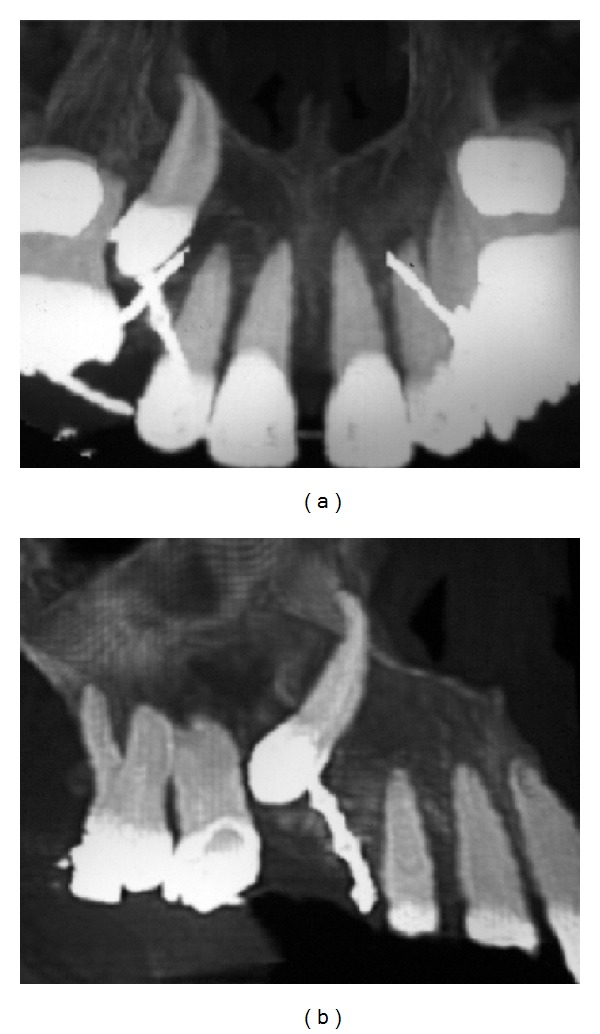
Right maxillary canine with large dilaceration root and orthodontic appliance for orthodontic traction.

**Figure 4:**
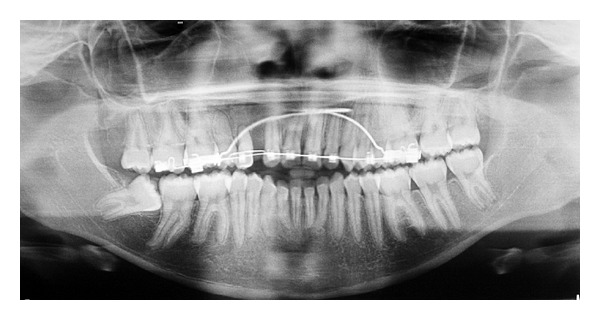
Final panoramic radiography: upper right canine positioned in the dental arch showing reduced root length.

Two years of orthodontic treatment were not able to change the impacted canine position. This behavior was determined because of the apical dilaceration in the canine's root (Figure 3). For this reason, a new surgical intervention was necessary, and the apicotomy of the dilacerated canine root was carried out [15, 16]. The technique consists of promoting an apical fracture of the impacted root's tooth. In this way, the periodontal area was reduced and mechanical retention was avoided by separating the dilacerated portion of the root and the tooth. The orthodontist could move the canine to a better position after the surgery and the upper canine retained was repositioned after five months of orthodontic movement (Figure 4). The pulp sensitivity test with cold responded positively and the tooth remained vital even in five years of followup. The canine root length was reduced as a consequence of the apicotomy.

## 3. Discussion

The canines occupy a strategic position in the upper arch developing an important role in the masticatory function. The lateral excursion with the canine-protected occlusion has been advocated as the best relationship for the mandibular excursion guide. This is due to the concave shape of the palatine surface of the canine, which is a good guide for the lateral movements, and due to the size of the canine root surface, which is able to resist the masticatory forces and provide a greater number of periodontal mechanoreceptors [18, 19]. Furthermore, it is easier to establish the deocclusion with orthodontic movement in a single tooth than with movement of a group of teeth. For these reasons, the canine is considered the most important tooth to be positioned in orthodontic treatment, which aims to promote a proper contour of the face and a final aesthetic smile [6].

Becker et al. recommend extraction of palatal canines severely impacted in height, when they are vertically positioned above the apices of the incisors [20]. The canine displacement in the present report was very severe, and the infection associated with the dentigerous cyst, as well as the apical dilaceration, was complicating factor. Despite that, the maintenance of the tooth was performed because the reduced bone volume in the affected canine could compromise the facial aesthetic at the end of the treatment. The eruption process promotes alveolar bone growth and, in this case, it helped the healing of the anterior maxilla.

The appropriate direction of eruption for a palatally impacted canine is essential for correcting the impaction and bringing the tooth to its correct position. It is important to remember that the presence of the periodontal ligament is necessary to allow tooth movement. This way, the main movement direction of the displaced tooth must be following its long axis, regardless of whether it will occur far from the correct position. The simple lateral traction of the tooth toward the edentulous alveolar ridge finds immediate resistance due to the compression of the canine crown against the adjacent palatine bone [10]. The consequence can be bone necrosis by compression and very slow movement, which can lead to a resorption process in the crown, and, thus, canine enamel damage can occur [21, 22]. These facts should be observed in order to minimize the orthodontic movement failure, as proposed by Becker et al. [14]. They concluded that the inaccuracy in diagnosis of location and orientation of impacted teeth and the failure to recognize anchorage demands were the major reasons for failure in the treatment of palatally displaced canines. However, in the same research, ankylosis was pointed out as a significant etiological factor that interfered in the success rate, corresponding to 32.4% of impacted canine failures. The ankylosis of the displaced teeth can be a consequence of the action of the low-speed drill during the surgery, chemical injury, and cervical periodontal ligament trauma by the magnitude and direction of orthodontic force [21]. Extensive surgery can be an additional factor compromising the cervical root area and promoting root resorption. The gaps formed by resorption are difficult to identify radiographically, and if the region is not repaired by cementoblasts, the bone can be deposited at the site, causing a lack of response to extrusive traction [20, 23].

Undoubtedly, the apicotomy allows traction of teeth with anatomical complications, but it is not always a simple procedure. The major difficulty in performing the apicotomy was sectioning the apical portion of the root without compromising the pulp tissue or with minimal injury to this tissue, as advocated by the technique [15, 16]. In the present case, the tooth was in a vertical position, located in the center of the alveolar crest, with the crown inclined for palatine. Moreover, the tooth was in a high position and the root was located more deeply, making it difficult to access the apical portion of the root. Despite the difficulty, it was possible to perform surgery successfully, which can be demonstrated by the position of the tooth in the dental arch and the positive response to the pulp vitality test. Importantly, the length of the canine root can be shortened, especially when the root dilaceration is extensive. This must be taken into account by the orthodontist, since the canine supports the load of the occlusion movement, as previously discussed.

## 4. Conclusion

Apicotomy is an effective technique for treating severe canine displacement and primary orthodontic traction failure of palatally displaced canines.
